# A Novel Integrated Biomarker for Evaluation of Risk and Severity of Coronary Atherosclerosis, and Its Validation

**DOI:** 10.3390/jpm12020206

**Published:** 2022-02-02

**Authors:** Victoria A. Metelskaya, Natalia E. Gavrilova, Maria V. Zhatkina, Elena B. Yarovaya, Oxana M. Drapkina

**Affiliations:** 1 National Medical Research Center for Therapy and Preventive Medicine, 101990 Moscow, Russia; yarovaya@mech.math.msu.su (E.B.Y.); odrapkina@gnicpm.ru (O.M.D.); 2 Scandinavian Health Center, 111024 Moscow, Russia; gavrilova_n@scz.ru; 3 Filatov City Clinical Hospital No 15, 111539 Moscow, Russia; mvzhatkina@gmail.com; 4 Department of Probability Theory, Faculty of Mechanics and Mathematics, Lomonosov Moscow State University, 119234 Moscow, Russia

**Keywords:** circulating biochemical markers, coronary atherosclerosis presence and severity, integrated biomarker (i-BIO), validation, visual markers

## Abstract

Objective: To assess the feasibility of a combination of biochemical and imaging parameters for estimation of risk and severity of coronary atherosclerosis (CA), and to verify the created integrated biomarker (i-BIO) on independent cohort. Methods: Two cohorts of patients admitted to the hospital for coronary angiography and ultrasound carotid dopplerography were enrolled into the study (*n* = 205 and *n* = 216, respectively). The extent of CA was assessed by Gensini Score (GS). Results: According to GS, participants were distributed as follows: atherosclerosis-free (GS = 0), CA of any stage (GS > 0), subclinical CA (GS < 35), severe CA (GS ≥ 35). Based on the analysis of mathematical models, including biochemical and imaging parameters, we selected and combined the most significant variables as i-BIO. The ability of i-BIO to detect the presence and severity of CA was estimated using ROC-analysis with cut-off points determination. Risk of any CA (GS > 0) at i-BIO > 4 was 7.3 times higher than in those with i-BIO ≤ 4; risk of severe CA (GS ≥ 35) at i-BIO ≥ 9 was 3.1 times higher than at i-BIO < 9. Results on the tested cohort confirmed these findings. Conclusions: The i-BIO > 4 detected CA (GS > 0) with sensitivity of 87.9%, i-BIO ≥ 9 excluded patients without severe CA (GS < 35), specificity 79.8%. Validation of i-BIO confirmed the feasibility of i-BIO > 4 to separate patients with any CA with sensitivity 76.2%, and of i-BIO ≥ 9 to exclude atherosclerosis-free subjects with specificity of 84.0%.

## 1. Introduction

Among the causes of premature death and disability, atherosclerosis-related cardiovascular diseases (ASCVDs), despite advances in their diagnostics and treatment, remain the leading cause worldwide, including in the Russian Federation [1,2,3].

Cardiovascular risk assessment, which is based on the identification of traditional risk factors, has a high prognostic value at the population level, but offers little information in terms of predicting individual risk [4]. Moreover, a significant number of acute coronary events are diagnosed in individuals with low or moderate risk calculated on the basis of epidemiological scores [5,6]. The effectiveness of noninvasive diagnostic tests (resting electrocardiography, echocardiography, computed tomography, or stress test) in evaluation of severity of ASCVD is not fully defined. For example, only 38% of patients without known heart disease who underwent elective invasive angiography had obstructive coronary heart disease [7], so better strategies for risk stratification are needed in order to increase the diagnostic power.

Understanding the molecular mechanisms of pathogenesis of atherosclerosis from subclinical to severe stages showed the high significance of biomarkers in ASCVD risks determination [8,9]. The increased interest in the study of cardiovascular risk markers in recent decades is largely due to advances in biomedicine, including both achievements in basic science and the development of new technologies to search for and study biomolecules [9,10,11]. In this connection, the problem of searching, validation and introduction into clinical practice of reliable, minimally invasive, and accessible to use markers, allowing assessment of the atherogenic potential of each individual at the early stages of the disease, i.e., before its clinical manifestations or development of complications, does not lose its relevance [11,12]. Indeed, the issue of using new biomarkers is widely discussed in the literature, including an analysis of the merits and demerits of both already known diagnostic and/or prognostic tools and the results of the latest developments using omics technologies [5,8,13,14].

Obviously, two different approaches might be used to improve the prediction of the risk of coronary atherosclerosis development and severity: either the use of additional biochemical markers or the use of noninvasive methods of imaging subclinical atherosclerotic vascular changes [15].

The analysis of the literature and our own data allowed us to conclude that it is expedient and relevant to study the possibility of applying a multi-marker approach to create diagnostic panels of biomarkers for individual risk assessment of ASCVDs and their complications. Indeed, novel complex or integrated biomarkers can include well-known metabolic parameters or combinations previously not considered for risk estimation. The clinical value of serum biomarkers for the diagnosis and prediction of manifestations of atherosclerosis has been assessed in numerous studies. A number of risk prediction models have been developed to assess ASCVD risk, but relatively modest prognostic power of individual biomarkers for risk prediction suggests that multiple biomarkers can be combined to improve their predictive power [13]. The multi-marker approach has been tested in several studies on circulating biomarkers [13,16,17,18].

An important step in the introduction of new biomarkers into clinical practice, in addition to the creation of new diagnostic panels themselves, is the assessment of their diagnostic/prognostic significance with the analysis of such characteristics as sensitivity and specificity, i.e., their validation [19]. Validation, or verification, of biomarkers involves their evaluation either in prospective follow-up of the original cohort (on which the biomarker was obtained) or in an independent cohort in a one-step analysis. Despite the large number of parameters claiming to be candidate biomarkers for cardiovascular risk assessment, few have passed the stages of clinical and analytical validation and have been recognized as real biomarkers [20]. Being in the era of personalized and preventive medicine, we have to understand that the main task of forming new biomarkers is to improve exactly the individual assessment of the probability of the presence and/or development of a disease and its prognosis.

Objective: To assess the feasibility of combination of biochemical and imaging markers for estimation of risk and severity of coronary atherosclerosis, and to verify a created integrated biomarker on an independent cohort.

## 2. Materials and Methods

The examined two cohorts consisted of patients admitted to the National Research Centre for Therapy and Preventive Medicine in Moscow for diagnostic coronary angiography: the initial cohort observed in 2012–2014 (*n* = 205, 66.0% males; mean age 62.8 ± 9.0 years), and the tested one observed in 2016–2019 (*n* = 216, 53.2% males; mean age 61.5 ± 10.7 years).

Inclusion criteria: consecutive inclusion of all patients over 18 years old who were admitted to the hospital for diagnostic angiography of coronary arteries. Exclusion criteria were: acute coronary syndrome within 6 months prior to the study; surgery within 6 months prior to the study; left ventricular ejection fraction below 40%; serious chronic or acute infectious diseases; chronic kidney diseases stages III and higher with glomerular filtration rate under 60 mL/min/1.73 m^2^); type I and II diabetes mellitus (level of glycosylated hemoglobin over 7.5%); familial hypercholesterolemia; neoplastic conditions; pregnancy and lactation.

All patients underwent coronary angiography according to the method Judkins (1967) using, as a rule, transfemoral access in the radiology operating setting with an angiographic unit Philips Integris Allura and General Electric Innova 4100. The procedure was performed considering the presence of at least one of the following causes: angina pectoris, history of myocardial infarction, heart rhythm disorders.

Location and extent of coronary artery lesions were assessed by Gensini Score (GS) [21]. Preliminary analysis of the most commonly used angiographic scoring systems revealed a good correlation between two angiographic scores (SYNTAX and GS) and quantitative assessment of coronary arteries (r = 0.87, *p* < 0.0001). GS had been chosen because according to our previous data [22], it appeared to be superior in estimation of severity of coronary atherosclerosis. The following GS cut-off points were used to estimate overall severity of vascular lesions: GS = 0 corresponds to unaffected coronary arteries; GS > 0 corresponds to the presence of coronary atherosclerosis of any stage; GS ≥ 35 corresponds to severe coronary atherosclerosis [22].

Atherosclerosis of carotid arteries was diagnosed by duplex B-mode scanning with color Doppler flow mapping 3–9 MHz linear transducer of PHILIPS iU22 ultrasound system in supine position with measuring carotid intima-media thickness (CIMT), determining the presence of atherosclerotic plaques (AP) in carotid arteries, and estimation of the degree (per cent) of stenosis. According to the experts [23], the values of CIMT < 0.9 mm have been chosen as the normal ones. The increase of CIMT was considered to be values from 0.9 to <1.3 mm, and AP criterion was indicated as CIMT > 1.3 mm or local CIMT increase by 0.5 mm (or by 50%) compared to the CIMT of the nearby vascular wall.

Anthropometric and clinical data included age, body mass index, waist circumference, and systolic and diastolic blood pressure. Blood was drawn from the cubital vein after 12–14 h of fasting prior to invasive angiography. Serum and plasma were obtained by low-speed centrifugation at 900× *g*, +4 °C; sample processing and storage were performed according to international guidelines [24]. Biochemical parameters were determined by standardized laboratory methods.

Statistical analysis was performed using the SPSS software version 20. Normal distribution of continuous variables was assessed using Shapiro–Wilk’s test. For normally distributed variables, the results were expressed as mean (M) ± standard deviation (SD) and for variables with significant deviations from the normal distribution were expressed as median (Me) and interquartile range (Q25–Q75). The differences between mean values were assessed using Student’s *t*-test. The nonparametric Mann–Whitney U-test was used to compare the medians of two independent groups. Multivariate logistic regression analysis with odds ratio calculations was used to screen for predictors of presence and severity of coronary atherosclerosis. The Receiver Operating Characteristic (ROC) curves were used to analyze the predictive power of integrated biomarker and to determine the optimal threshold values for the AP number and carotid stenosis degree, as well as for fibrinogen and adiponectin levels. The thresholds for all the rest of the parameters have been chosen according to the corresponding Guidelines [23,25,26]. *p* values < 0.05 were considered statistically significant.

## 3. Results

The first step of our study was devoted to creation of a novel integrated biomarker for estimation of risk of coronary atherosclerosis presence and severity. The total initial cohort was split into patients with unaffected coronary arteries (GS = 0; *n* = 39) and those with any stage of coronary atherosclerosis (GS > 0; *n* = 166). The extent of coronary lesion was estimated by Gensini Score [21]; all patients were split into two groups with no/subclinical atherosclerosis (GS < 35; *n* = 112) and severe coronary atherosclerosis (GS ≥ 35; *n* = 93). No differences in major anthropometric characteristics were found between groups. A wide range of circulating parameters was analyzed, including lipoprotein profile, markers of insulin-dependent glucose utilization, inflammatory and hemostasis markers, and parameters of visceral adipose tissue metabolism (Table 1).

Preliminary analysis of various mathematical regression models included structure parameters of the arterial wall, biochemical markers, and their combinations allowed us to select the most significant variables (listed in the Table 2) which together represent a combined or integrated biomarker called i-BIO. For each parameter incorporated into i-BIO, the points were assigned depending on cut-off values indicating deviations from the normal conditions, and detailed scoring of these parameters was carried from minimal (no changes) to maximal (presence of marked changes).

Thus, imaging parameters included in i-BIO were: CIMT (≤0.9; >0.9 mm), the number of AP (<3; ≥3), and the degree of carotid stenosis (≤45; >45%). Metabolic parameters incorporated into i-BIO included levels of triglycerides (TG) (≤1.7; 1.8-1.9; ≥2.0 mmol/L), glucose (≤5.5; 5.6–6.0; 6.1–6.9; ≥7.0 mmol/L), fibrinogen (≤4.0; >4.0 g/L), high sensitive C-reactive protein (hsCRP) (<1.0; 1.0–2.9; ≥3.0 mg/L), and adiponectin (<8.0; ≥8.0 µg/mL) as shown in Table 2. The sum of points calculated for each patient represents individual i-BIO value.

**Figure 1:**
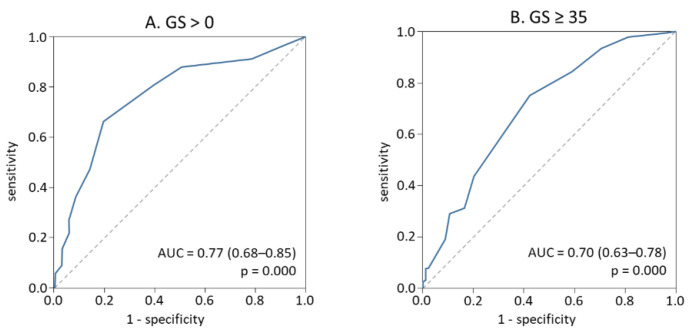
ROC-curve analysis for the prediction of coronary atherosclerosis presence (**A**) or severity (**B**) by i-BIO in the initial cohort.

To assess the discriminative power of i-BIO, we performed the ROC curve analysis and used the corresponding areas under curve (AUC) to determine the sensitivity and specificity of the i-BIO (Figure 1). Two threshold values of i-BIO to assess the risk of coronary atherosclerosis and its severity were found. The threshold i-BIO value over 4 points for patients with coronary atherosclerosis of any stage (GS > 0) was proposed for detection of this disease presence with a sensitivity of 87.9% and specificity of 50.0% (Figure 1A). Risk of coronary atherosclerosis (GS > 0) among patients with i-BIO > 4 points was 7.3 times higher than for patients with i-BIO ≤ 4 points (OR = 7.3; 95% CI 3.2–16.4, *p* = 0.0001). In patients with GS ≥ 35 the threshold for i-BIO ≥ 9 points was proposed to be optimal for prediction of severity of coronary atherosclerosis with a relatively low sensitivity of 43.8% but with rather high specificity of 79.8% (Figure 1B). Risk of severe coronary atherosclerosis (GS ≥ 35) at i-BIO ≥ 9 points was 3.1 times higher than for patients with i-IBIO < 9 points (OR = 3.1; 95% CI 1.6–5.8, *p* = 0.001).

The i-BIO was validated on an independent (tested) cohort of patients in whom the presence or absence of coronary atherosclerosis was also verified by coronary angiography, and changes in the carotid arteries were detected by duplex scanning. The cohort included 216 patients; their biochemical characteristics are presented in Table 3.

**Figure 2:**
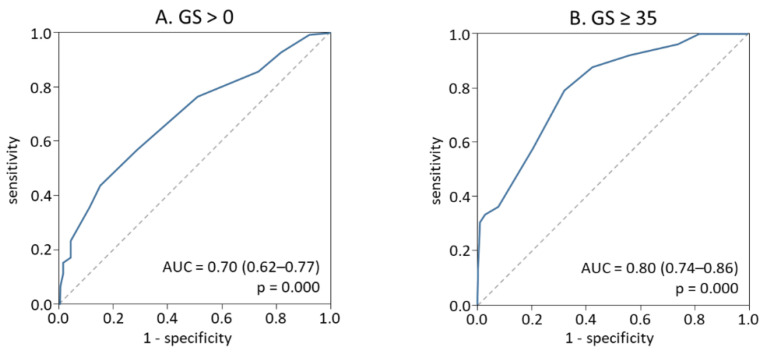
ROC-curve analysis for the prediction of coronary atherosclerosis presence (**A**) or severity (**B**) by i-BIO in the tested cohort.

To assess the risk of presence and severity of coronary atherosclerosis in this tested cohort, the same thresholds for i-BIO as for initial cohort were used: i-BIO > 4 or BIO ≥ 9, respectively. Figure 2A demonstrates the results of ROC curve analysis for the feasibility of i-BIO to discriminate between atherosclerosis-free subjects (GS < 0) and patients with coronary atherosclerosis of any severity (GS > 0). At i-BIO > 4, risk of any stage atherosclerosis detection was 3.1 times higher than in subjects having i-BIO ≤ 4: OR = 3.1 (95% CI 1.7–5.7; *p* = 0.0002) with sensitivity of 76.2% and specificity–49.3%.

Verified marker i-BIO ≥ 9 allows us to separate patients with multivessel coronary lesions and clinical manifestations (GS ≥ 35) from those who did not have severe atherosclerosis with sensitivity of 50.0% and specificity of 84% (Figure 2B). Risk of severe atherosclerosis at i-BIO ≥ 9 was 5.3 times higher than in patients with i-BIO < 9 (OR = 5.3; 95% CI 2.75–10.03; *p* = 4 × 10^−7^).

## 4. Discussion

This study was undertaken to examine a set of clinical and metabolic parameters and their combinations in order to analyze their potential impact on early development of atherosclerotic lesions in coronary arteries to predict the presence and severity of coronary atherosclerosis. We have generated a novel cumulative multi-marker—integrated biomarker, or i-BIO, composed of several imaging and circulating biochemical parameters for non-invasive detection of coronary atherosclerosis and evaluation of its severity. According to the data obtained, i-BIO appeared to be valid and allowed statistically significant separation of both atherosclerosis-free subjects (GS = 0) from patients with any signs of atherosclerosis according to coronary angiography data (GS > 0) and patients without changes on coronary angiography from those with severe atherosclerosis of coronary arteries (GS ≥ 35).

Atherosclerosis is a systemic process, and this prompted us to analyze biochemical parameters of function or dysfunction of the major metabolic systems involved into pathogenesis of this disease including serum lipoprotein profile, markers of insulin-dependent glucose utilization, inflammatory and hemostatic markers, and parameters of visceral adipose tissue metabolism.

Biomarkers play an essential role in biomedical research, in daily clinical practice, and in drug development, which should be considered one of the priority areas of translational medicine [27,28,29,30]. In this regard, research to find new biomarkers, to evaluate their analytical characteristics, and to confirm their significance seems to be a very relevant task. Moreover, before being introduced into biomedical research any new marker requires verification (validation) either in a prospective study or in cross-sectional studies on independent cohorts.

Now it is becoming increasingly clear that a single-biomarker approach has limited predictive value, while integrated combined biomarkers have a definite advantage over the measurement and analysis of individual parameters and can be used as additional tools to further individual stratification of the patients at risk. The multi-marker approach has been tested in a number of studies [13,16,17,18,29,30,31]. In particular, it has been shown that when the initial low correlation between a single marker and the disease or its risk factors increases significantly when they are combined; this approach is very promising [28,32,33]. Several algorithms incorporating multiple markers were used for prediction of coronary heart disease risk [34]. Indeed, integrated biomarkers may add incremental value to other criteria that include traditional risk factors, and should be considered in those patients who require particular management decisions but the physician lacks sufficient information to assess conventional risk factors in these patients. The use of new technologies, including proteomics, metabolomics, lipidomics and advanced structural and functional imaging, enable to detect a number of promising new biomarker candidates [35,36,37].

Along with this, a certain place is given to the approach used in our study, when combined multi-markers are formed on the basis of generally used in practical healthcare studies and measurements, including non-invasive duplex scanning of carotid arteries and quantification of circulating metabolites included in this biomarker. Another promising approach to analyze a wide set of parameters for new effective scales construction is machine learning, commonly called artificial intelligence [38]. Using machine learning approaches and computational modeling, several studies have been undertaken for the early diagnostics and prediction of coronary artery disease status and progression, for the prediction of atherosclerotic plaque growth [39], or to assess the risk level of diabetes mellitus [40].

In other words, new biomarkers and/or their complexes may well occupy a niche both as auxiliary non-invasive diagnostic tools and as new potential molecular targets. Thus, the study of various combinations of clinical and demographic, imaging, and biochemical/metabolic/genetic parameters available for practical health care, as well as the establishment of integrated biomarkers and assessment of their predictive ability, allowed us to propose multi-marker diagnostic panels for non-invasive (low invasive) personalized detection of coronary atherosclerosis and its severity.

Our study has some limitations. We lack the follow up data for the patients and thus cannot estimate the outcome and evaluate predictive power of the i-BIO for the purpose of calibration and reclassification. However, we verified our data on an independent cohort similar to the initial one; moreover, a follow-up study on the initial cohort is in progress.

## 5. Conclusions

In conclusion, the novel i-BIO suggested in our study includes imaging characteristics derived from carotid ultrasound dopplerography (CIMT, AP number, and carotid stenosis) and circulating blood tests (serum levels of triglycerides, glucose, fibrinogen, hsCRP, and adiponectin). i-BIO values over 4 points detected patients with any stage of coronary atherosclerosis (GS > 0) with sensitivity of 87.8%, while i-BIO ≥ 9 points allows to exclude patients free from severe coronary atherosclerosis (GS < 35) with specificity of 79.8%. Validation of i-BIO on the independent cohort confirmed the feasibility of i-BIO > 4 to separate patients with coronary atherosclerosis of any stage (GS > 0) with sensitivity of 76.2%, and to exclude subjects without atherosclerosis (GS < 35) with specificity of 84.0% with i-BIO ≥ 9. Thus, a composite panel of routinely available imaging and blood tests is useful for assessment for coronary atherosclerosis and estimation of its severity.

## Figures and Tables

**Table 1:** Circulating biochemical parameters of the patients from the initial cohort.

Characteristics Means ± SD; Median (Q25–Q75)	GS = 0 (*n* = 39)	GS > 0 (*n* = 166)	GS < 35 (*n* = 112)	GS ≥ 35 (*n* = 93)
TC, mmol/L	5.5 ± 1.3	5.1 ± 1.2	5.2 ± 1.2	5.1 ± 1.3
LDL-C, mmol/L	3.7 ± 1.3	3.1 ± 1.1 ^a^	3.3 ± 1.0	3.2 ± 1.2
HDL-C, mmol/L	1.1 ± 0.3	1.0 ± 0.3 ^a^	1.0 ± 0.3	1.0 ± 0.3
Triglycerides, mmol/L	1.6 (1.2–2.1)	1.6 (1.2–2.2)	1.6 (1.2–2.1)	1.6 (1.2–2.2)
apo AI, g/L	1.70 ± 0.29	1.55 ± 0.28	1.60 ± 0.32	1.54 ± 0.25
apo B, g/L	1.02 ± 0.24	0.89 ± 0.24 ^a^	0.95 ± 0.23	0.87 ± 0.26 ^b^
hsCRP, mg/L	2.8 (1.1–5.7)	2.8 (1.3–5.8) ^a^	2.4 (1.1–5.1)	3.3 (1.5–6.6) ^b^
Fibrinogen, g/L	3.7 (3.2–4.1)	3.6 (3.2–4.1)	3.6 (3.2–4.1)	3.7 (3.3–4.2)
Glucose, mmol/L	5.7 ± 1.1	6.0 ± 1.5	5.9 ± 1.5	6.0 ± 1.4
Insulin, μU/mL	10.1 (7.4–15.0)	10.5 (7.3–14.5)	10.2 (7.2–14.3)	10.7 (7.5–14.9)
Adiponectin, μg/mL	9.2 (7.1–11.8)	7.8 (5.4–11.5) ^a^	8.0 (5.8–11.8)	7.8 (5.5–11.4)
Leptin, ng/mL	33.5 (15.9–49.5)	19.4 (10.6–32.5)	22.9 (10.6–36.9)	19.1 (11.9–33.8)

TC—total cholesterol; LDL-C—low-density lipoprotein cholesterol; HDL-C—high-density lipoprotein cholesterol; hsCRP—high-sensitive C-reactive protein; apo–apolipoprotein; *p* < 0.05: a—between GS = 0 and GS > 0; b—between GS < 35 and GS ≥ 35.

**Table 2:** Integrated biomarker (i-BIO).

Parameters	Points
Sex	0—female 1—male
Ultrasound carotid dopplerography parameters: CIMT, mm AP, n Extent of carotid stenosis, %	0—CIMT ≤ 0.9, AP < 3, Extent of carotid stenosis ≤45 1—CIMT > 0.9, AP < 3, Extent of carotid stenosis ≤45 2—CIMT ≤ 0.9, AP ≥ 3, Extent of carotid stenosis ≤45 3—CIMT > 0.9, AP ≥ 3, Extent of carotid stenosis ≤45 4—CIMT ≤ 0.9, AP < 3, Extent of carotid stenosis >45 5—CIMT > 0.9, AP < 3, Extent of carotid stenosis >45 6—CIMT ≤ 0.9, AP ≥ 3, Extent of carotid stenosis >45 7—CIMT > 0.9, AP ≥ 3, Extent of carotid stenosis >45
Triglycerides, mmol/L	0—TG < 1.7 1—1.7 ≤ TG < 2.0 2—TG ≥ 2.0
Glucose, mmol/L	0—Glucose ≤ 5.5 1—5.5 < Glucose ≤ 6.0 2—6.0 < Glucose < 7.0 3—Glucose ≥ 7.0
Fibrinogen, g/L	0—Fibrinogen ≤ 4.0 1—Fibrinogen > 4.0
hsCRP, mg/L	0—hsCRP < 1.0 1—1.0 ≤ hsCRP < 3.0 2–hsCRP ≥ 3.0
Adiponectin, µg/mL	0—Adiponectin ≥ 8.0 1—Adiponectin < 8.0

CIMT—carotid intima-media thickness; AP—atherosclerosis plaque; hsCRP—high-sensitive C-reactive protein; TG—triglycerides.

**Table 3:** Circulating biochemical parameters of the patients from the tested cohort.

Characteristics Means ± SD; Median (Q25–Q75)	GS = 0 (*n* = 73)	GS > 0 (*n* = 143)	GS < 35 (*n* = 144)	GS ≥ 35 (*n* = 72)
TC, mmol/L	4.5 ± 1.2	4.2 ± 1.1	4.6 ± 1.08	3.8 ± 0.9 ^b^
LDL-C, mmol/L	2.6 ± 0.9	2.6 ± 0.9	2.7 ± 0.9	2.2 ± 0.8 ^b^
HDL-C, mmol/L	1.2 ± 0.3	1.1 ± 0.3	1.2 ± 0.3	1.0 ± 0.3 ^b^
Triglycerides, mmol/L	1.4 (1.0–1.9)	1.3 (1.0–1.8)	1.3 (0.9–1.9)	1.4 (1.1–1.8)
apo AI, g/L	1.57 ± 0.32	1.47 ± 0.29 ^a^	1.58 ± 0.29	1.36 ± 0.26 ^b^
apo B, g/L	0.86 ± 0.24	0.88 ± 0.23	0.89 ± 0.24	0.84 ± 0.22
hsCRP, mg/L	2.3 (0.9–5.4)	3.0 (1.6–6.3) ^a^	2.8 (1.1–4.8)	4.5 (2.1–10.1) ^b^
Fibrinogen, g/L	4.30 (3.7–5.2)	4.70 (4.1–5.5) ^a^	4.30 (3.8–5.1)	4.90 (4.4–5.7) ^b^
Glucose, mmol/L	5.9 ± 1.4	6.6 ± 1.8 ^a^	6.1 ± 1.4	6.9 ± 2.0 ^b^
Insulin, μU/mL	8.85 (6.1–12.5)	11.0 (7.6–15.9) ^a^	9.4 (6.0–13.8)	11.0 (8.2–17,5) ^b^
Adiponectin, μg/mL	7.7 (5.9–10.6)	5.1 (5.6–11.2)	8.3 (6.2–11.4)	6.8 (4.7–10.4) ^b^
Leptin, ng/mL	19.4 (5.8–57.3)	16.9 (4.8–58.2)	21.4 (5.2–70.7)	10.5 (4.0–37.2) ^b^

TC—total cholesterol; LDL-C—low-density lipoprotein cholesterol; HDL-C—high-density lipoprotein cholesterol; hsCRP—high-sensitive C-reactive protein; apo—apolipoprotein; *p* < 0.05: a—between GS = 0 and GS > 0; b—between GS < 35 and GS ≥ 35.
